# Prospective Associations between Emotion Dysregulation and Fear-Potentiated Startle: The Moderating Effect of Respiratory Sinus Arrhythmia

**DOI:** 10.3389/fpsyg.2016.00652

**Published:** 2016-05-06

**Authors:** Antonia V. Seligowski, Daniel J. Lee, Lynsey R. Miron, Holly K. Orcutt, Tanja Jovanovic, Seth D. Norrholm

**Affiliations:** ^1^Department of Psychology, Northern Illinois UniversityDeKalb, IL, USA; ^2^Department of Psychology, Auburn UniversityAuburn, AL, USA; ^3^Department of Psychiatry and Behavioral Sciences, Emory University School of MedicineAtlanta, GA, USA; ^4^Trauma Recovery Program, Atlanta Veteran Affairs Medical CenterDecatur, GA, USA

**Keywords:** emotion regulation, fear-potentiated startle, respiratory sinus arrhythmia, trauma, physiology

## Abstract

**Background:** Emotion dysregulation has been implicated in the negative outcomes following trauma exposure. A proposed biomarker of emotion dysregulation, respiratory sinus arrhythmia (RSA), has demonstrated associations with trauma-related phenomena, such as the fear-potentiated startle (FPS) response. The current study aimed to examine the prospective association between emotion dysregulation and RSA and FPS several years following trauma exposure.

**Methods:** Participants were 131 women exposed to a campus mass shooting on February 14, 2008. Pre-shooting emotion dysregulation was assessed in 2006–2008. Startle response, measured by orbicularis oculi electromyography (EMG), and RSA were gathered during an FPS paradigm conducted from 2012 to 2015.

**Results:** No significant associations among emotion dysregulation, RSA, and FPS emerged among the full sample. However, emotion dysregulation predicted FPS during both acquisition (*r* = 0.40, *p* < 0.05) and extinction (*r* = 0.57, *p* < 0.01) among individuals with high resting RSA.

**Conclusions:** Findings suggest that pre-shooting emotion dysregulation is a potent predictor of FPS several years following potential trauma exposure, and this association varies by RSA level. Results emphasize the importance of examining autonomic regulation in the association between emotion dysregulation and recovery from trauma exposure.

## Introduction

Emotion dysregulation, or difficulty modulating physiological, behavioral, and subjective components of emotional responding (Linehan, 1993; Linehan et al., 2007), has been implicated in the development and maintenance of several forms of fear-based psychopathology, including panic, phobias, and posttraumatic stress disorder (PTSD; Kring and Bachorowski, 1999; Aldao et al., 2010; Bardeen et al., 2013). Replicated cross-sectional findings have established a clear correlation between emotion dysregulation and these disorders (Aldao et al., 2010; Seligowski et al., 2015). Additionally, Bardeen et al. (2013) demonstrated that pre-trauma emotion dysregulation predicted PTSD symptoms following trauma exposure. However, the prospective association between emotion dysregulation and specific aspects of fear learning involved in the development and maintenance of fear-based disorders remain unexamined to date.

Fear learning is theorized to play a central role in inhibiting recovery from traumatic event exposure (Foa and Kozak, 1986). In fear learning, individuals exposed to a fearful or traumatic event develop conditioned responses (CR) whereby conditioned stimuli (CS; e.g., a green jeep) previously paired with an unconditioned stimulus (US; e.g., an explosion) evoke the same responses in the absence of the US. These CSs (e.g., sights, smells, sounds) evoke strong autonomic nervous system (ANS) responses characterized by increases in sympathetic nervous system (SNS) activity coupled with withdrawn parasympathetic nervous system (PNS) activity (see Pole, 2007 for a review). As a result, individuals may learn to fear particular CSs associated with the fearful experience (e.g., all green vehicles) and exhibit a CR even in the presence of safety.

Previous research has demonstrated that individuals with fear-based disorders tend to experience deficits in fear learning. Specifically, these individuals demonstrate exaggerated fear potentiated startle (FPS) responses to both danger cues (CS associated with US) and safety cues (CS not associated with US) compared to those without PTSD (Jovanovic et al., 2009, 2010, 2012; Norrholm et al., 2011; Sijbrandij et al., 2013). In addition, Norrholm et al. (2011) found that individuals with PTSD demonstrated greater FPS responding to a previously reinforced CS during extinction compared to those without PTSD. Consistent with the theorized role of fear learning in inhibiting recovery from traumatic event exposure, these findings establish a clear distinction between fear conditioning among those who do and do not recover from trauma exposure. Additionally, research has demonstrated that individuals with panic disorder and social phobia exhibit larger FPS responses and worse fear discrimination (evidenced by increased responding to a safety cue) compared to healthy controls (Grillon et al., 1994; Larsen et al., 2002; Lissek et al., 2009). Collectively, these results establish an association between deficits in fear learning and fear-based psychopathology.

While research has yet to examine emotion dysregulation in association with fear learning, previous studies have demonstrated that fear learning is associated with cardiac vagal control, a proposed biomarker of emotion dysregulation (Beauchaine, 2001). Cardiac vagal control is the influence of the vagus nerve on the heart as mediated through respiration (Berntson et al., 1993, 1997). Specifically, Polyvagal theory (Porges et al., 1994; Porges, 2007) conceptualizes cardiac vagal control as an indicator of one's ability to maintain homeostasis (i.e., regulate) by activating the PNS (i.e., the ability of an individual's vagus nerve to stimulate the sinoatrial node, resulting in slowed heart rate). Common measures of cardiac vagal control include heart rate variability (HRV) and respiratory sinus arrhythmia (RSA; Berntson et al., 1993, 1997). Polyvagal theory is particularly relevant to fear learning because it addresses how classical conditioning and physiological reactivity interact to form fear responses, including both fear acquisition (SNS) and fear inhibition (PNS). Higher cardiac vagal control has been associated with lower startle responding to affective stimuli, as well as to both predictable and unpredictable threat of electric shock (Ruiz-Padial et al., 2003; Gorka et al., 2012). A later study by Gorka et al. (2013) found that individuals with higher cardiac vagal control demonstrated greater reductions in startle over time (i.e., greater habituation) in comparison to those with lower cardiac vagal control. This association has also been observed among individuals with differing levels of anxiety sensitivity, as well as panic disorder. Using a FPS paradigm, Melzig et al. (2009) found that students with high cardiac vagal control exhibited greater startle habituation than those with low cardiac vagal control. In addition, individuals with panic disorder demonstrated lower cardiac vagal control and greater startle magnitudes compared to controls with high cardiac vagal control (Melzig et al., 2009). Lastly, Pappens et al. (2014) examined cardiac vagal control and startle responding in an interoceptive FPS paradigm using obstructed breathing as the US. The authors found that individuals with high cardiac vagal control demonstrated greater fear inhibition and fear extinction compared to those with lower cardiac vagal control (Pappens et al., 2014).

One critical limitation of the existing literature is a dearth of prospective designs. This scarcity of longitudinal research is limiting for a number of reasons, perhaps the most important of which is the inability to identify risk factors for subsequent deficits in fear learning. The current study sought to examine emotion dysregulation in predicting FPS responding among a trauma-exposed sample using a prospective design. Specifically, it was hypothesized that poor pre-trauma emotion dysregulation would predict greater startle responding and poor fear inhibition during a FPS paradigm several years post-trauma. In addition, given that cardiac vagal control is proposed as a physiological indicator of emotion dysregulation, we sought to examine baseline cardiac vagal control as a predictor of these variables as well. Similar to our expectations regarding emotion dysregulation, it was hypothesized that low cardiac vagal control during fear conditioning would predict greater startle responding and poor fear inhibition during extinction.

## Method

### Procedure and participants

Data were collected from women who completed multiple waves of a longitudinal study approved by the Northern Illinois University (NIU) Institutional Review Board. Between August 2006 and February 14, 2008, students taking an Introductory Psychology course received partial course credit for completing an initial assessment for a longitudinal investigation of risk factors for sexual revictimization (*N* = 1045 with 885 participants consenting to follow-up contact). Participants were required to be women over the age of 18 years old and fluent in English. Trauma history or posttraumatic stress symptomatology were not selection criteria. Measures included at this time point were computer administered in individual sessions of ~1 h and participants received partial course credit.

Following the NIU campus shooting on February 14th, 2008, the NIU Trauma Study was launched in order to examine outcomes following the event. Women previously enrolled in the longitudinal investigation of sexual revictimization were recruited to participate in a multi-wave longitudinal study examining postshooting adjustment. Seventeen days following the shooting, all eligible participants determined to be current NIU students (*n* = 812 of the 885 participants who consented to follow-up contact) were invited to participate in the postshooting adjustment study via e-mail. Of these participants, 691 (85.10%) completed the initial postshooting assessment and were recruited for subsequent postshooting assessments (postshooting survey assessments are not included in the present study; see Orcutt et al., 2014 for detail regarding 30-month postshooting recruitment and survey assessment). Between October 2012 and February 2015, participants who completed the preshooting assessment and agreed to participate in the multi-wave postshooting follow-up were recruited via e-mail, phone call, and mail contact to participate in an FPS experimental session. Women who were currently pregnant were excluded from the FPS portion of data collection (*n* = 1). Of the 691 eligible participants, 131 (18.96%) completed the 30-min online pre-FPS survey (for which participants were paid $25), and the 120-min FPS experimental session. Given that participants also provided blood and saliva samples during the experimental portion, compensation was $100 for the FPS session. The time interval between preshooting and FPS assessments ranged from 62 to 96 months (*M* = 76.72, *SD* = 7.37). Time elapsed between the shooting and completion of the FPS assessment ranged from 61 to 83 months (*M* = 70.69, *SD* = 5.51).

Participants in the final sample (*N* = 131) ranged from 23 to 34 years old (*M* = 25.5, *SD* = 1.9) at FPS data collection. In terms of race, 100 participants (76.3%) self-identified as European American, 22 (16.8%) as African American, 5 (3.8%) as “Other,” and 2 (1.5%) as Asian American; 2 (1.5%) chose not to respond. Eight participants (6.1% of the final sample) endorsed a separate item indicating they identified with Hispanic or Latina ethnicity.

### Self-report measure

#### Difficulties in emotion regulation scale (DERS)

The DERS (Gratz and Roemer, 2004) is a 36-item measure of several aspects of emotion dysregulation. Each item is rated on a 5-point Likert scale based on how often respondents believe each item pertains to them, ranging from 1 (*almost never*) to 5 (*almost always*). For the current study, a total DERS score was used, with higher scores indicating greater emotion dysregulation. The DERS has demonstrated good psychometric properties in prior research (Gratz and Roemer, 2004). Cronbach's alpha in the current study was 0.83.

### Psychophysiological assessment

Startle reflex magnitude was measured using the electromyography (EMG) module of the BIOPAC MP150 for Windows (Biopac Systems, Inc., Aero Camino, CA) as in previously published work (e.g., Norrholm et al., 2011). Data were filtered, rectified, and smoothed using AcqKnowledge software (Biopac Systems, Inc., Aero Camino, CA) and exported for statistical analyses. The EMG signal was sampled at a rate of 1000 Hz and filtered with low- and high-frequency cutoffs at 28 and 500 Hz, respectively. The maximum amplitude of the eye-blink muscle contraction 20–200 ms after presentation of the startle probe was used as a measure of the acoustic startle response. The eye-blink component of the acoustic startle response was measured by EMG recordings of the right *orbicularis oculi* muscle with two 5-mm Ag/AgCl electrodes positioned 1 cm below the pupil of the right eye and 1 cm below the lateral canthus. Impedance levels were < 6 kΩ for each participant. The startle probe was a 108-dB (A) SPL, 40-ms burst of broadband noise with near instantaneous rise time, delivered through headphones.

Heart rate was measured using the electrocardiogram (ECG) module of the Biopac system at a sampling rate of 1 kHz. One 5-mm Ag/AgCl electrode was placed on participants' chest above the right clavicle and another electrode was placed on participants' left radius. A respirometer was placed around participants' natural waist to measure respiration. Cardiac vagal control was measured using RSA. RSA was calculated from the raw Biopac recordings (i.e., ECG and respiration) using MindWare EMG and HRV software (MindWare Technologies, Inc.,). MindWare was used to identify ECG R-waves and R-R intervals (i.e., the time between hear-beats) and detect improbable R-waves, which were then manually inspected and corrected. Settings for high and low frequency bands were based on standard recommendations for RSA data (respiratory band of 0.15–0.40 Hz; Task Force, 1996).

The FPS protocol consisted of two phases: fear acquisition and fear extinction. During both phases, colored shapes were used as CSs and were presented on a computer monitor for 6 s using SuperLab software (Cedrus, Inc.). In the fear acquisition phase (20 min in duration), one stimulus (CS+) was paired with an aversive US while another stimulus (CS−) was not. Fear acquisition began with a habituation phase consisting of startle noise probes presented alone and non-reinforced conditioned stimuli. This was followed by three conditioning blocks with four trials of each type (CS+ reinforced, CS−, and startle noise probe alone, NA) for a total of 12 trials per block and 36 total trials. Startle probes were delivered on each trial type. The aversive US, a 250-ms airblast with an intensity of 140 p.s.i. directed at the larynx, was delivered 500 ms after the acoustic probe. This US has been used previously and reliably produces robust FPS (e.g., Norrholm et al., 2011). In all phases, the inter-trial intervals were randomized to be 9–22 s in duration. Following fear acquisition, participants were given a 10-min break, during which they completed payment forms for the study. Participants then underwent the fear extinction phase (25 min in duration) where the CS+ was no longer paired with the US. The extinction phase consisted of 6 blocks with four trials of each type (CS+, CS−, and NA) for a total of 12 trials per block and 72 total trials.

### Statistical analysis

All statistical analyses were conducted in SPSS version 22.0. Data were first inspected for quality and normality. Of the full sample, 16 participants (12.21%) had missing data. Univariate outliers were identified as beyond two interquartile ranges from the median and treated as missing. After removing outliers, only a small portion of data (6.68% of data points) was missing. Missing data was handled using multiple imputation (MI). Following guidelines by Bodner (2008), a conservative 24 datasets were imputed and pooled for all analyses.

After examination of descriptive statistics, bivariate correlations were conducted to examine study hypotheses. Specifically, FPS to the CS+ during blocks 2 and 3 of acquisition (FPS-Acq), as well as was FPS to the CS+ during all blocks of extinction (FPS-Ext) was examined in relation to pre-shooting emotion dysregulation (DERS) and RSA during the FPS paradigm. Only FPS during blocks 2 and 3 of acquisition were used because learning the distinction between the CS+ and CS− is taking place in block 1 and is therefore less meaningful (i.e., sufficient time has not passed for learning to take place; Norrholm et al., 2011; Jovanovic et al., 2013). FPS to the CS+ was calculated using difference scores [(startle magnitude in the presence of the CS+ in each conditioning block) − (startle magnitude to NA); Jovanovic et al., 2005].

## Results

**Figure 1:**
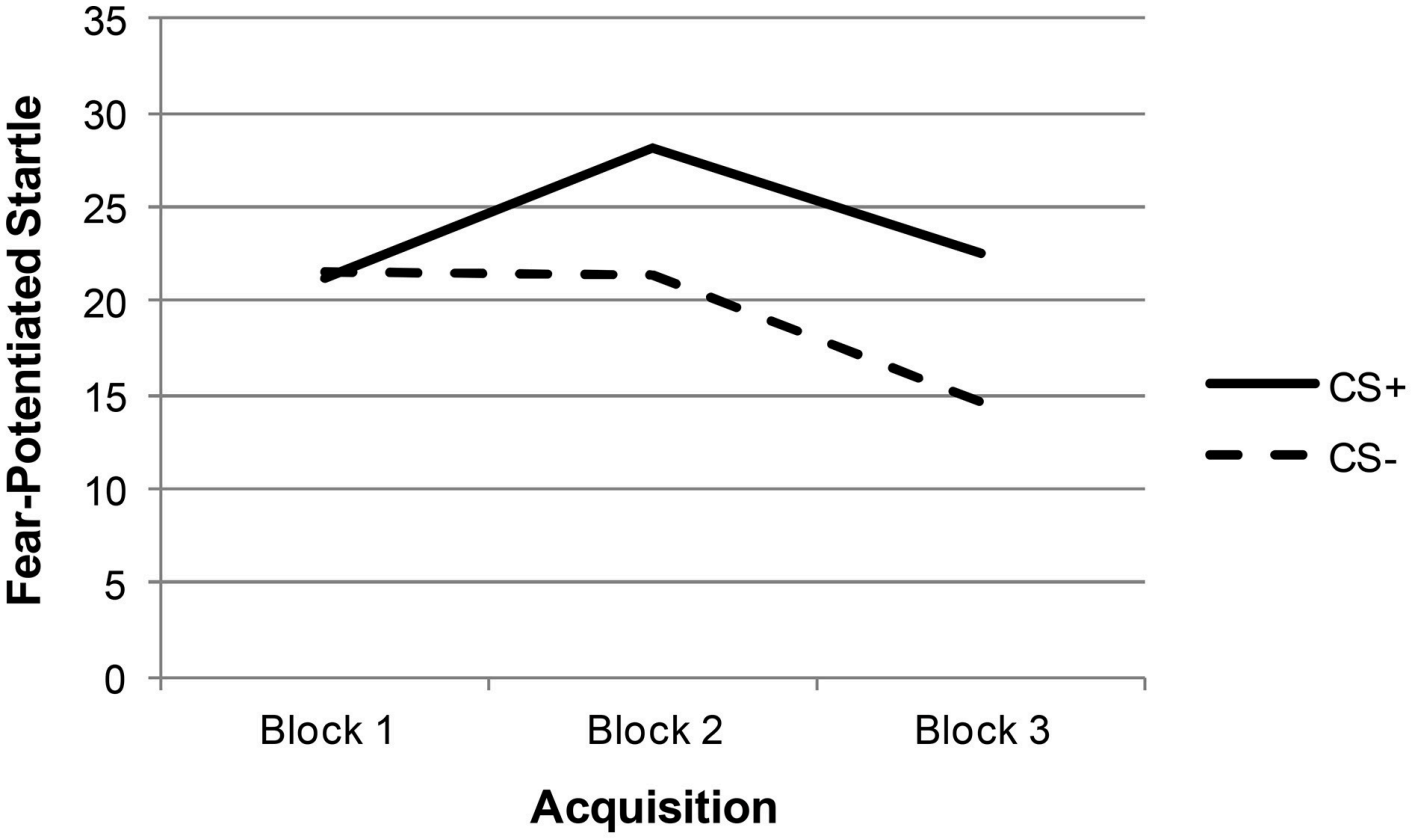
**Acquisition fear-potentiated startle among total sample**.

**Figure 2:**
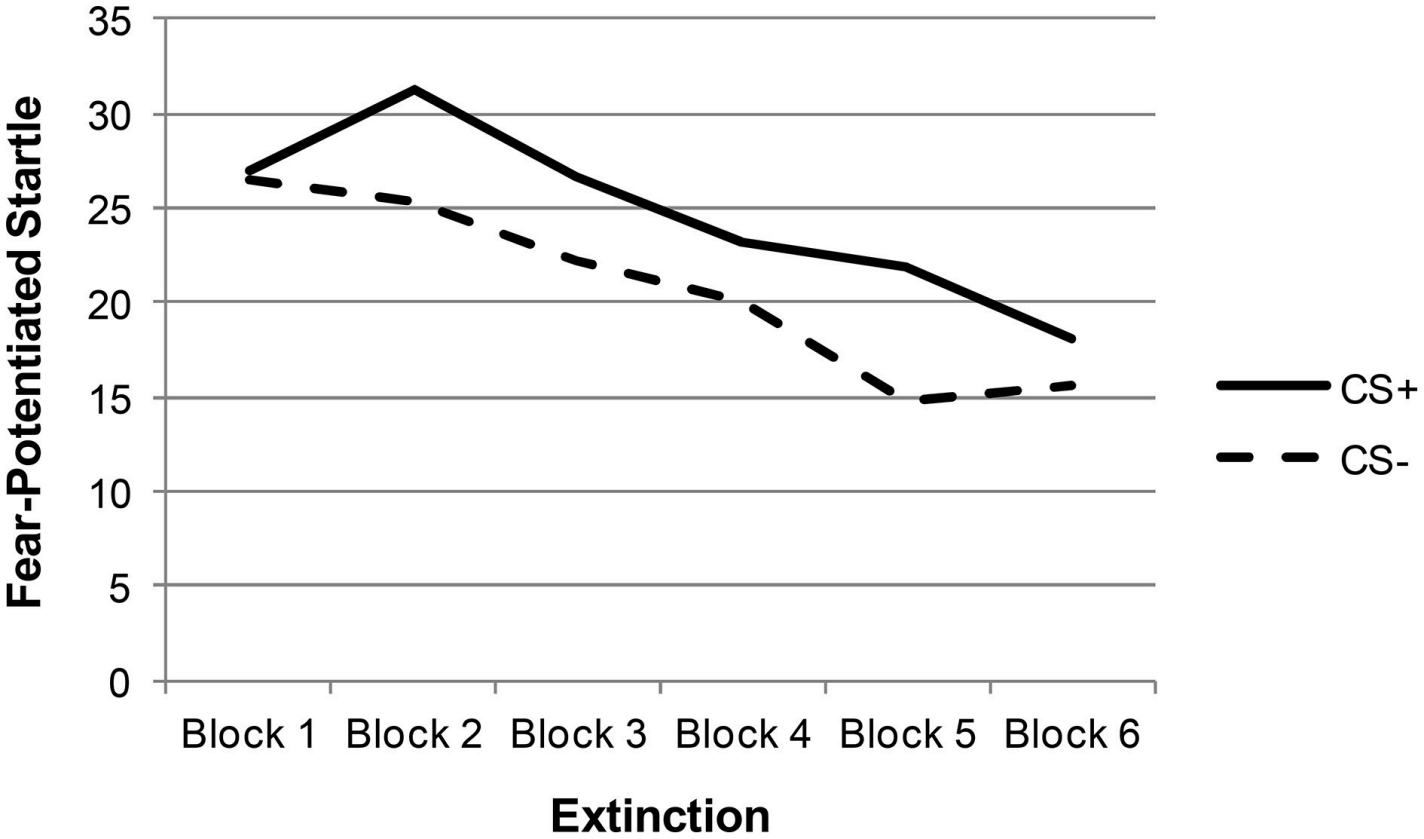
**Extinction fear-potentiated startle among total sample**.

Descriptive statistics and zero-order correlations between variables are provided in Table 1. A repeated measures ANOVA indicated that the effect of stimulus type (CS+ vs. CS−) was significant across late acquisition blocks, suggesting that participants were successfully fear conditioned [*F*_(1, 129)_ = 5.97, *p* = 0.016; see Figure 1]. Another repeated measures ANOVA indicated that the effect of stimulus type was also significant across extinction blocks: *F*_(1, 127)_ = 9.74, *p* = 0.002. In addition, there was a significant linear effect of block in extinction, suggesting that both CS+ and CS− scores decreased over time [an indication that fear was successfully extinguished; *F*_(1, 127)_ = 16.62, *p* < 0.001; see Figure 2].

**Table 1:** **Descriptive statistics and zero-order correlations among included variables**.

	**1**	**2**	**3**	**4**
1. DERS-Total	−	−	−	−
2. Tonic RSA	0.07	−	−	−
3. FPS-Acq	0.09	0.02	−	−
4. FPS-Ext	0.10	−0.05	0.48^*^	−
Mean	85.31	6.61	15.69	17.83
SD	13.25	1.08	20.39	17.57
Range	55−119	3.91−10.32	−46.97 to 76.25	−15.67 to 73.11

*DERS, Difficulties in Emotion Regulation Scale; RSA, Respiratory Sinus Arrhythmia; FPS-Acq, FPS response during blocks 2 and 3 of acquisition; FPS-Ext, FPS response during all blocks of extinction*.

* *p < 0.01*.

**Figure 3:**
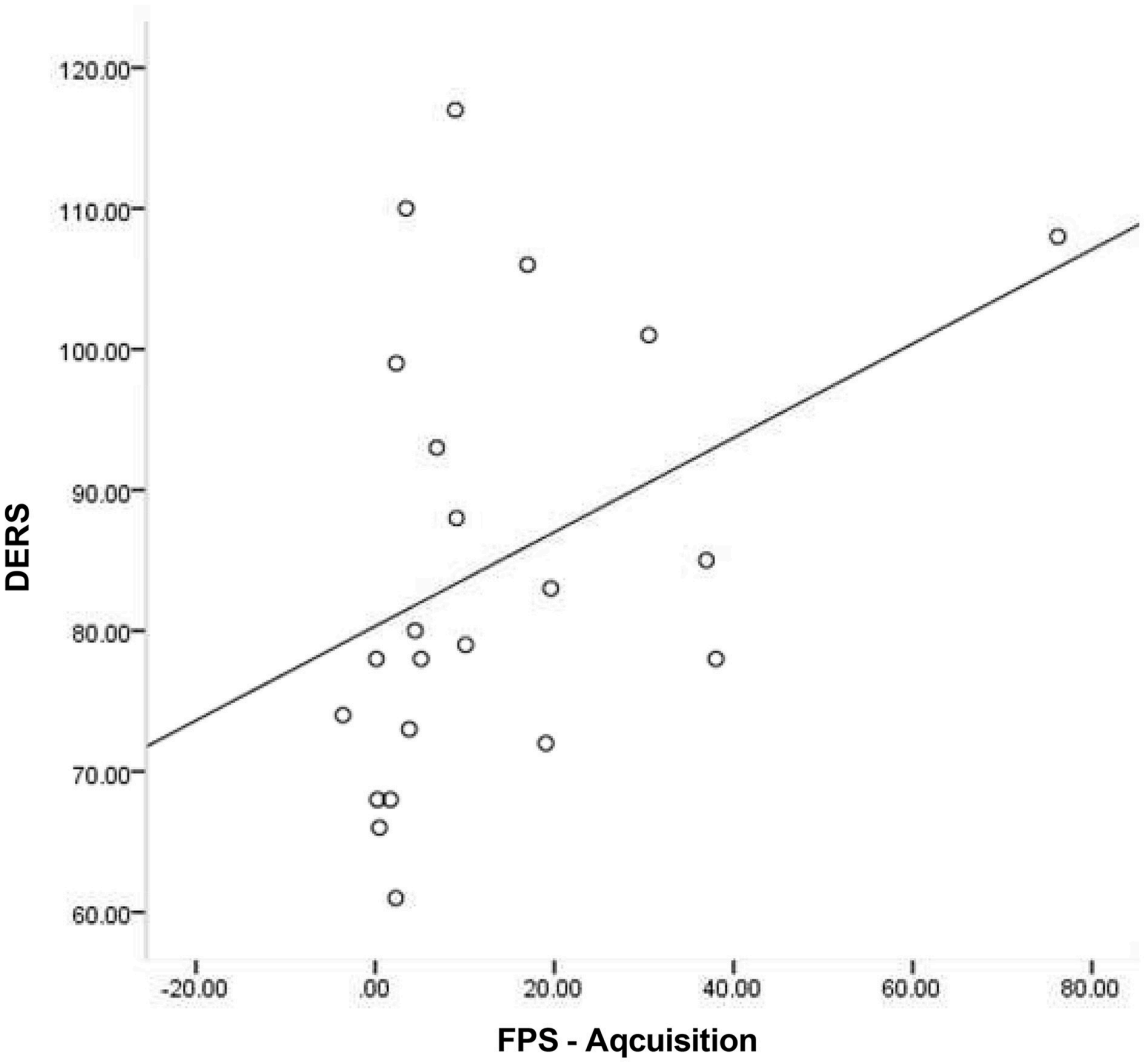
**Scatter plot of DERS and FPS-Acquisition among those with high RSA**.

**Figure 4:**
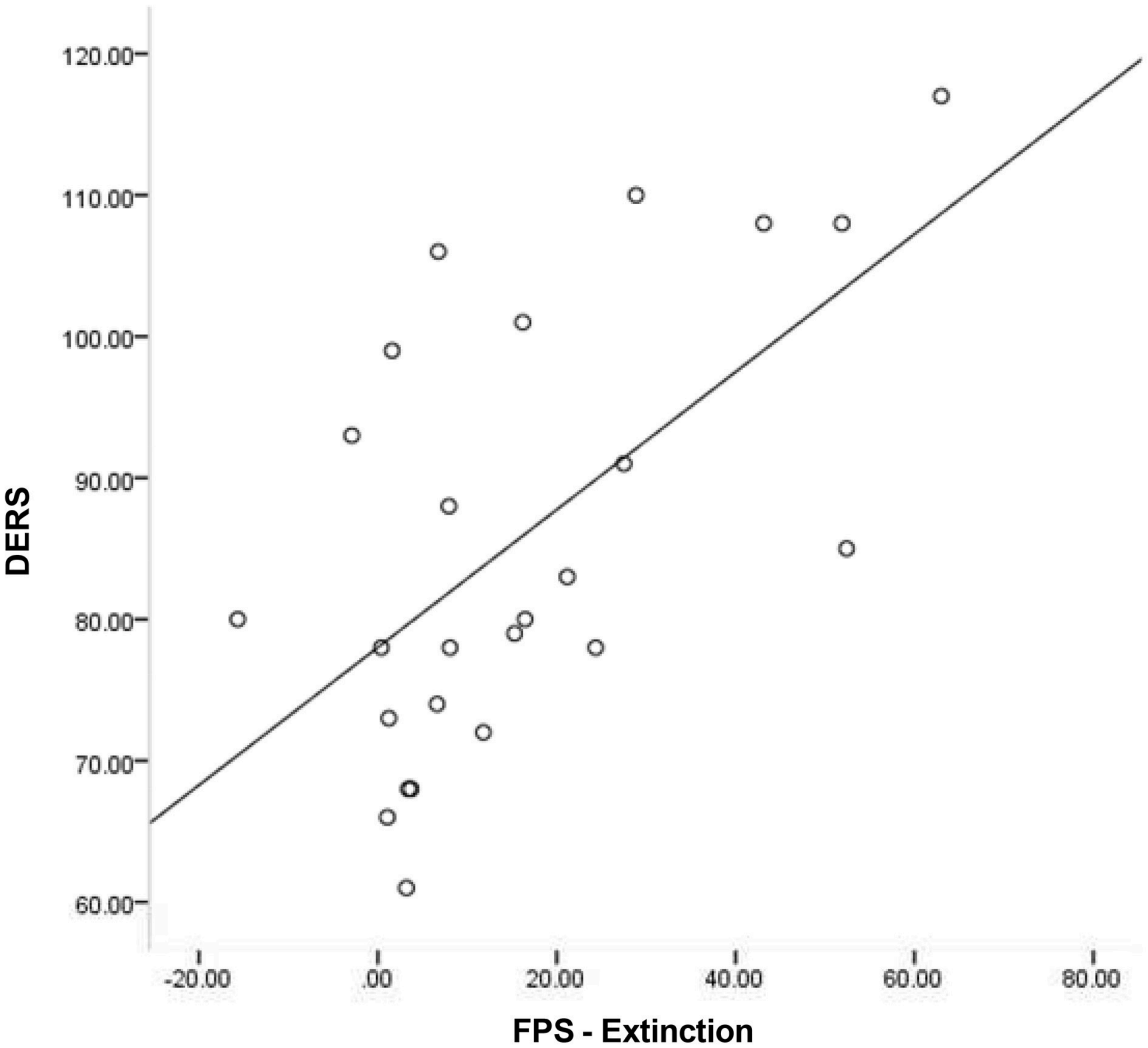
**Scatter plot of DERS and FPS-Extinction among those with high RSA**.

Given that neither emotion dysregulation nor RSA were associated with the FPS variables at the bivariate level, testing of initial hypotheses with regressions was not warranted. Review of scatter plots indicated that associations between emotion dysregulation and FPS appeared to differ by RSA level. Accordingly, subsequent *post-hoc* analyses were conducted using an extreme-groups approach, whereby participants were sorted into high (+1 SD) and low (−1 SD) resting RSA groups (*n*s = 28 and 16, respectively). Mean RSA in the high group was 8.36 (*SD* = 0.66, range = 2.61), while mean RSA in the low group was 4.99 (*SD* = 0.43, range = 1.60). The bivariate associations between pre-shooting emotion dysregulation and FPS were examined separately within each of these groups. Within the low RSA group, pre-shooting emotion dysregulation was not significantly associated with startle magnitude during late acquisition or extinction periods. However, pre- shooting emotion dysregulation was significantly associated with startle magnitude during both late acquisition (*r* = 0.40, *p* < 0.05) and extinction (*r* = 0.57, *p* < 0.01) periods among the high RSA group. The direction of the effect was such that greater emotion dysregulation was associated with greater fear-potentiated startle response during both periods. See Table 2 and Figures 3, 4 for summaries of these associations.

**Table 2:** **Correlations among emotion dysregulation and fear-potentiated startle among high and low RSA Groups**.

	**Low RSA (*n* = 16)**			**High RSA (*n* = 28)**		
	**1**	**2**	**3**	**1**	**2**	**3**
1. DERS	−			−		
2. FPS-Acq	−0.07	−		0.40^*^	−	
3. FPS-Ext	−0.24	0.59^*^	−	0.57^**^	0.61^**^	−
*M*	82.1	8.6	15.2	85.5	13.3	15.9
*SD*	13.7	20.2	17.3	14.8	18.4	19.4
Range	48.0	91.1	59.2	56.0	79.8	78.7

*DERS, Difficulties in Emotion Regulation Scale; RSA, Respiratory Sinus Arrhythmia; FPS-Acq, FPS response during blocks 3 and 4 of acquisition; FPS-Ext, FPS response during all blocks of extinction*.

* p < 0.05;

** *p < 0.01*.

## Discussion

Emerging research suggests that cardiac vagal control is related to physiological indicators of fear learning and fear inhibition (i.e., FPS; Ruiz-Padial et al., 2003; Gorka et al., 2012, 2013; Pappens et al., 2014). While lower cardiac vagal control has been posited as a biomarker of emotion dysregulation, no previous studies have examined the role of cardiac vagal control in the association between self-reported emotion dysregulation and fear learning. The current study examined the prospective effects of pre-shooting emotion dysregulation on FPS response several years later. In addition, we sought to examine the predictive effect of cardiac vagal control on FPS. Participants were a cohort of women who were exposed to a campus mass shooting in 2008.

Surprisingly, neither pre-shooting emotion dysregulation nor RSA was significantly associated with FPS responding during acquisition or extinction periods among the full sample and emotion dysregulation was not associated with RSA at the zero-order level. These results suggest that ability to modulate emotional responding and cardiac vagal control may not function as the salient protective factors for everyone as they are theorized to. Rather, emotion dysregulation and cardiac vagal control may have a more nuanced association with deficits in fear learning. When examined separately among high and low cardiac vagal control groups, emotion dysregulation was prospectively associated with FPS, but only among individuals with high cardiac vagal control. These findings suggest autonomic regulation may play a critical role in the prospective impact of emotion dysregulation on post-trauma fear learning.

The finding that the prospective association between emotion dysregulation and FPS was only significant among individuals with higher RSA suggests a potential extension of the existing conceptualization of the link between cardiac vagal control and emotion dysregulation. Both emotion dysregulation and its proposed biomarker, cardiac vagal control, are associated with important psychological phenomena (e.g., negative affect, anxiety, depression, PTSD; Beauchaine, 2001; Demaree et al., 2004; Aldao et al., 2010; Bardeen et al., 2013). Results of the current study suggest that higher cardiac vagal control may be necessary for emotion dysregulation to prospectively influence fear learning. Specifically, results suggest that individuals with better ability to regulate emotion prior to trauma exposure experienced less FPS several years later, but only among individuals with high resting RSA. Accordingly, the ability to achieve regulatory goals of modulating physiological, behavioral, and subjective components of emotional responding may only function as a protective factor following trauma exposure for individuals with greater cardiac vagal control.

Results of the current study support previous research suggesting that pre-trauma emotion dysregulation is predictive of post-trauma sequelae (Bardeen et al., 2013). However, this is the first study to demonstrate that self-reported emotion dysregulation is predictive of FPS responding, in particular. It is worth noting that these associations were found among variables separated by ~7 years in measurement, thus increasing the robustness of study findings. An additional strength of the current study is its use of a prospective longitudinal design, which allows for the examination of emotion dysregulation prior to a common fateful event (i.e., the campus mass shooting). Future studies of emotion dysregulation and FPS may benefit from the consideration of separate groups based upon autonomic functioning.

The current study has several limitations. First, the current study included mostly individuals who identified as White and non-Hispanic. Therefore, generalization of results to other racial and ethnic groups is limited. Second, the current study consisted of exclusively female participants. Results may not generalize to men or to mixed sex samples, as sex differences in RSA have been reported previously (e.g., Overbeek et al., 2012). Third, emotion dysregulation was assessed using self-report. Although the majority of emotion regulation research to date has been conducted using self-report measures, these instruments have come under criticism (see Lee et al., 2015). Accordingly, future research should examine these associations using alternative measurement approaches. Fourth, an important limitation to note in the current study is the observed association among a small group of participants (*n* = 28). The size of this group certainly limits the degree to which these findings can be interpreted. As the first study to identify this association, these findings warrant replication among larger and more diverse samples. Fifth, the length of time between pre-trauma emotion dysregulation measurement and FPS/RSA measurement is both a strength and limitation of the current study. Although this length of time speaks to the salience of emotion dysregulation as a predictor of subsequent fear learning, it is not possible to control for all of the other experiences, changes, and development that occurs over this much time. Accordingly, future research should examine this association at both short and long intervals to parse out the short- and long-term associations between these variables. Additionally, findings from the current study should be interpreted within the limitations inherent to the nature of the study design. As trauma onset could not have been predicted, pre-shooting FPS responding and RSA are unknown in this sample. Accordingly, it is not possible to examine the direction of the observed effects. For example, it is possible that pre-shooting differences in FPS account for pre-shooting DERS or the degree to which FPS changed from pre- to post-shooting. Accordingly, the direction of the effects among emotion dysregulation, RSA, and FPS cannot be examined. Given the inability to examine trauma exposure within an experimental design, the current findings should be interpreted as providing some insight into the association between pre-trauma emotion dysregulation and subsequent FPS, but causality is far from established. Future research should examine the temporal associations between RSA, emotion dysregulation, and FPS among trauma exposed populations, ideally shortly after trauma exposure (e.g., Shalev and Freedman, 2005).

To conclude, this is the first known study of the prospective effects of emotion dysregulation on later FPS responding. Findings suggest that autonomic functioning may be a key difference factor in this association, and that it may serve a protective role in post-trauma sequelae. Future research on emotion dysregulation and FPS may benefit from the examination of individuals with high vs. low RSA as separate groups.

## Ethic statement

The authors assert that all procedures contributing to this work comply with the ethical standards of the relevant national and institutional committees on human experimentation and with the Helsinki Declaration of 1975, as revised in 2008.

## Author contributions

All authors listed, have made substantial, direct and intellectual contribution to the work, and approved it for publication.

### Conflict of interest statement

The authors declare that the research was conducted in the absence of any commercial or financial relationships that could be construed as a potential conflict of interest.
